# Antibiotic Prescription for Endodontic Treatment: General Dentist Knowledge + Practice in Shiraz

**Published:** 2011-05-15

**Authors:** Mohammad Reza Nabavizadeh, Safoora Sahebi, Ilnaz Nadian

**Affiliations:** 1. Department of Endodontics, Dental School, Shiraz University of Medical Sciences, Shiraz, Iran.; 2. Dentist, Private Practice, Shiraz, Iran.

**Keywords:** Antibiotic, Endodontics, General Practitioners, Prescription

## Abstract

**INTRODUCTION:**

Diseases of the dental pulp and periapical tissues are chiefly caused by microorganisms. Antibiotics are used in some endodontic cases; however, successful cases can predominantly be achieved by mechanical and chemical cleaning of the canal or surgical intervention.

**MATERIALS AND METHODS:**

The aim of this study was to determine the knowledge of General Dental Practitioners (GDPs) in Shiraz in respect to antibiotic prescriptions during and after endodontic treatment. A one-page questionnaire was sent to 200 active general dentists. Of the 120 surveys returned, 93 were accepted. The data were analyzed using t-test, Chi-square, ANOVA and Fisher’s Exact Test.

**RESULTS:**

Only 29% of dentists had full knowledge (correct answers to all questions) of antibiotic prescription protocols in pulpal and periapical disease. Amoxicillin 500 mg capsule was the drug of choice of dentists. Total of 42% of GDPs had full knowledge of antibiotic prescription protocols for persistent or systemic infections cases. GDPs more recently qualified had slightly greater knowledge compared to GDPs with experience; however, this difference was not significant. Also, there was no significant difference between genders.

**CONCLUSION:**

General practitioners’ knowledge about antibiotics seems inadequate and further education is recommended to update the practitioners.

## INTRODUCTION

Bacterial resistance to antimicrobials has been an ongoing problem for clinicians ever since the discovery of antimicrobial agents; this is due to bacterial species ability to develop resistance to all antibacterial agents shortly after they had been marketed and used [1].

There is sufficient evidence that shows a significant relationship between increase in antimicrobial resistance and antimicrobial utilization, with higher resistance levels in bacteria isolated from areas with high antibiotic utilization compared with those from areas with low antibiotic utilization [2].

Antibiotics can be responsible for various adverse effects, including drug interaction, nausea, gastrointestinal upsets, potentially fatal allergic reactions and antibiotic associated colitis [3]. Antibiotics are being used inappropriately by dentists in different clinical conditions. The primary treatment of endodontic infections is to establish and maintain surgical drainage and to remove the cause of the infection. Despite usefulness of antibiotics, in most cases successful treatment can be achieved by mechanical and chemical cleaning of the root canal [3][4][5][6][7][8][9][10].

Palmer et al. showed that 12.5% of general dental practitioners (GDPs) prescribed antibiotics for teeth with acute pulpitis.

Antibiotic prescription has been reported for 30.3% of GDPs in cases of time limitations, and 47.3% when a precise diagnosis was not impossible. The most commonly prescribed antibiotic was amoxicillin [11]. Another study showed that 61.48% of GDPs prescribe penicillin V as drug of choice for endodontic infections. In case of allergy to penicillin, the alternative choice was clindamycin (57.3%) and erythromycin (26.65%) [12]. A study in Yazd showed that 60.6% of dentist prescribed penciling V as primary selected drug for endodontic infections. In case of allergy to penicillin, erythromycin was administered in 70.49% of cases. Dentist prescribed antibiotic in cases of irreversible pulpits (13.8%), necrotic pulps without pain and swelling (21.53%) and sinus tract (47.69%) [13].

Zadik and Levin evaluated the influence of geographic location of graduation (Israel, Eastern Europe, Latin America) on decision making regarding management of dental caries, periapical lesions and antibiotic prescribing routines. They found that significantly more Latin American graduates prescribed antibiotics following endodontic treatment, retreatment, and third molar extractions [14].

The analysis of national data available from health solutions in Wales showed that dental prescribing in Wales accounted for 9% of total antibacterial prescribing in primary care in 2008. Penicillin and metronidazole constituted the bulk of antibiotics prescribed by the dentists [15]. A study on Belgian dentists found that antibiotic was often prescribed in the absence of fever (92.2%) and without any local treatment (54.2%). The most frequent diagnosis for which antibiotic were prescribed was periapical abscess (51.9%) [16]. Another study in Australia showed that generally there was an appropriate level of knowledge of antibiotic prescription. However, there was a tendency toward over prescription and a lack of knowledge of the incidence of adverse reactions, development of multi-resistant strains and prophylaxis against bacterial endocarditis [17].

Murti and Morse in a study conducted in Fiji found that prescription of antibiotic increased with clinical experience and there was a moderate level of knowledge regarding specific indications for antibiotic prescription both therapeutically and prophylactically. In addition, approximately one third of respondents stated antibiotic resistance as a problem in Fiji and 40% reported experiencing some form of antibiotic resistance in clinical practice [18].

The knowledge and practice of Shiraz general practitioners has not been assessed yet. The aim of this study was to determine GDPs’ knowledge of antibiotic prescribing in Shiraz during and after endodontic treatment.

## MATERIALS AND METHODS

This descriptive research was approved by an ethical committee in Shiraz. A questionnaire (Table 1) was designed to investigate general dental practitioners’ knowledge of antibiotics prescription protocols. The questionnaire was first evaluated in a pilot study. Modifications were made to the questionnaire to reach an acceptable level in validity and reliability. It was then sent to the general dental practitioners in Shiraz. The questionnaire comprised of questions relating to age, gender, work experience and year of graduation. The questionnaire investigated practitioners’ knowledge of the indications for prescribing antibiotics for a number of systemic clinical signs that may be associated with a dental infection. The clinical signs chosen were fever and malaise, evidence of systemic spread, diffused swelling, and difficulty in swallowing. GDPs were also asked whether some clinical conditions required antibiotics and their choice of treatment if any. These clinical conditions were acute pulpitis, acute apical abscess, chronic apical abscess with sinus tract, chronic apical periodontitis. Also a number of factors that can influence antibiotic prescriptions were investigated. The questionnaire asked whether patient’s expectation of prescribing antibiotics, two session root canal treatment, one long session root canal treatment, and retreatment might be the reason for prescribing antibiotics. The next part of the questionnaire assessed knowledge on the medical conditions and dental procedures that may require prophylactic antibiotics. The dental procedures were all root canal treatments, pre and post endodontic surgeries; the medical conditions included HIV+, HBS+, non-controlled diabetes, congenital heart diseases, mitral valve prolapse and patients who have had prosthetic joint in the past 2 years or those with a history of cancer and radiotherapy.

**Table 1:**
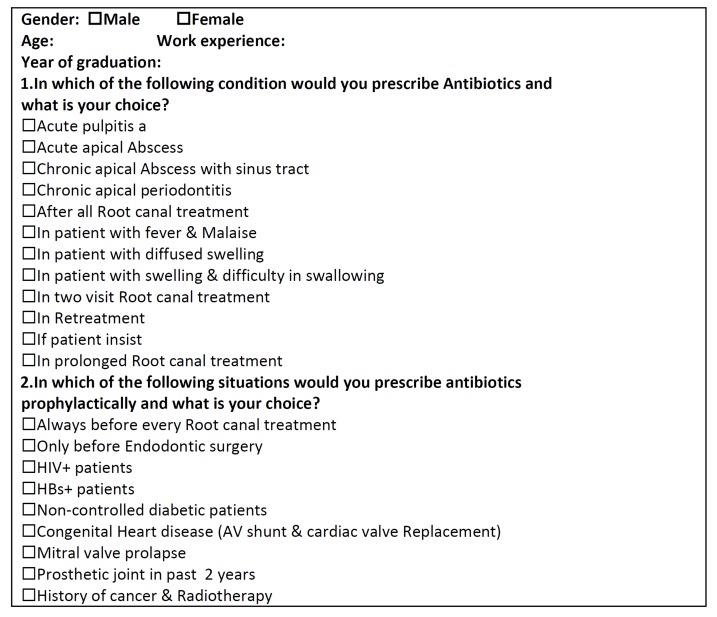
Questionnaire

The study sample was selected from dentists in Shiraz accredited by Islamic Republic of Iran Medical Council. To determine the sample volume a Kokaran formula was used and 10% of GDPs (n=93) were selected through a cluster sampling. Shiraz was divided into five geographic zones. Stratified clustering sampling technique was used which incorporated 5 stratified zones, for each of which a cluster of active general dentists were randomly recruited. Overall, 200 GDPs were recruited to this study and a one page questionnaire was sent to them (Table 1). A total of 120 of GDPs fully answered and returned the questionnaires. Questionnaires which had less than 30% answered questions were excluded; therefore, atotal of 93 questionnaires remained for analysis. Data were analyzed using SPSS software version Mac OS X and presented using descriptive measures.

## RESULTS

The overall response rate to questionnaires was 46.5 % (93 fulfilled acceptance criteria). The demographics of respondents are described in Table 2. Male and female dentists correctly answered 68% and 63% of the questions, respectively. GDPs with fewer years of practice have slightly higher knowledge compared to more experienced GDPs (Table 3).

**Table 2:** Description of respondents

**Male**	57%
**Female**	43%
**Mean Age(y)**	36
**Mean Years in Practice**	9

**Table 3:** Percentage of correct answers corresponding to cears of practice

**Years of Practice **	**Correct Answers (%)**
**0-2 **	72
**3-5**	69
**6-10 **	60
**Over 10 years**	64****

ANOVA test revealed no significant differences between respondents in relation to sex and number of years in practice. Only 1% of respondents answered all the questions of prophylactic antibiotic coverage for medically compromised patients correctly. The drug of choice for these cases was 2g Amoxicillin (80.4%). Other prophylactic drugs that were prescribed by dentists are summarized in Table 4.

**Table 4:** Antibiotics that were prescribed by dentists prophylactically

**Antibiotic**	**%**
**2g Amoxicillin**	80.4
**3g Amoxicillin**	6.2
**1g Amoxicillin**	5.3
**Amoxicillin+Metronidazole**	2.1
**2g Penicillin V**	0.9
**6g Amoxicillin**	0.9
**2g Cephalexin**	0.2
**Refer to Specialist **	4.1****

The greatest number of antibiotic prescriptions was written for acute pulpits (80.6%) and acute periapical abscess (74.2). Overall, 11% of respondents always prescribed antibiotics after root canal therapy (11%) (Table 5). For chronic periapical lesions and chronic periapical abscess plus sinus tracts, 73.1% and 58% of respondents prescribed antibiotics, respectively.

**Table 5:** Conditions and percentage of antibiotic prescription

**Conditions**	**Prescribe Antibiotics (%)**
**Acute Pulpitis**	80.6
**Acute Periapical Abscess**	74.2
**Chronic Periapical Abscess with Fistula**	58
**Chronic Periapical Lesion**	73.1
**Always after all root canal treatment**	11
**If the patient insist**	14
**Two session root canal treatment**	16
**One long session root canal treatment**	35
**Retreatment **	42

Overall, only 20% of GDPs had full knowledge of antibiotic prescription for pulpal and periapical diseases. Amoxicillin 500mg was the drug of choice (57.6%) in all correct and incorrect cases. Based on the correct answers, 42% of GDPs had full knowledge of antibiotic prescription. In cases of persistent or systemic infection (i.e. fever, swelling, lymphadenopathy, trismus, or malaise), penicillin IM was the drug of choice (26.6%), followed by amoxicillin 500mg (25.3%).

## DISCUSSION

The overall response rate from 200 GDPs in this study was 46.5%. Other similar surveys published have reported response rates of 35% and 31% [19][20]; therefore the overall response rate was considered an acceptable rate of return for the surveys. The questionnaire design in this study included variety of information about GDPs’ knowledge and behavior in antibiotic prescription in relation with their experience and gender.

GDPs that had graduated more recently had greater knowledge compared to GDPs with more experience. These results concurred with a similar study performed by Gutierrez et al. [21]. This is probably due to the more recent and fresh evidence-based information of recent graduates. Only 20% of GDPs correctly answered all questions.

Antibiotics are not indicated for acute pulpitis; however, 80.6% of Shiraz GDPs prescribed antibiotics; whereas Palmer et al. showed that 12.5% of British GDPs prescribed antibiotics [11]. Another two studies conducted in the USA showed approximately 16% antibiotic prescription [12][22], however one study had only surveyed endodontist specialists [12] which was similar to Gatewood et al’s. in 1990 [22]. A study conducted in Yazd on GDP prescription habits found much lower rate of unnecessary prescription (13.84%) [13].

Antibiotics are not indicated and will not assist cases where the pulp is still vital and there are no signs of local or systemic infection/involvement [23].

Even in cases of a necrotic pulp, chronic apical periodontitis with fistula or a chronic periapical lesion in a healthy patient, there is no indication for antibiotic use and treatment should be limited to non-surgical root canal therapy. In this study 58% of GDPs prescribed antibiotics which were very different to percentages quoted in other studies 35.7% [12] and 18.85% [24]. In the Yazd study, 47.69% of dentists still prescribed antibiotics [13]. The proper treatment for irreversible pulpitis cases is debridement of the root canal space. Non-surgical root canal therapy without antibiotic is usually adequate to treat irreversible pulpitis, acute pulpitis and chronic apical periodontitis and draining sinus tract. The pulpal circulation is compromised in these cases and systemic antibiotic will not reach therapeutic concentrations in the pulp. Removing the source of the infection by performing thorough non-surgical root canal therapy will usually allow healing of periradicular lesion. Analgesics however are indicated for periapical and pulpitis pain [25].

GDPs knowledge of dental infections with systemic signs like fever, malaise, difficulty in swallowing was inadequate; only 42% answered all the related questions correctly. Various antibiotics were prescribed in these cases which were similar to reports of other studies [25][26][27][28]. However, the most prescribed drug was penicillin IM (26.6%). Tabrizizadeh and Alijani showed that the drug of choice in dental infections was Penicillin V (60.6% of dentists prescribed it) [13], concurring with the study conducted on American endodontists who mostly prescribed penicillin V (61.48%) in dental infections with systemic signs [12]. However, the antibiotic of choice in these cases should have been penicillin IM which was prescribed by 26.6% of GDP’s. In this study, 42% of GDPs prescribed antibiotics for root canal retreatments, whereas, this was 15.38% in Yingling et al. study [12].

According to the present study, general dentists in Shiraz use antibiotics inappropriately; this could lead to problems such as drug resistance, resistant microorganisms and other side effects.

It would appear from this study that GDPs knowledge about the use of antibiotics in general practice is far from ideal. This mirrors general medical practice where studies have shown that decision making in antibiotic therapy requires improvement. Rational prescribing based on a thorough evidence-based knowledge is essential. Effective communication between microbiologists and practitioners and the publication of prescribing guidelines and protocols could help to achieve this [29]. Moreover correct educational intervention may also be effective [30]. An audit of antibiotic prescribing in dental practice showed that there was a reduction in the number of prescriptions following the introduction of guidelines [31].

The use of clinical audit and computers along with other tools to increase knowledge of antibiotic prescribing and improve patient care should not be underestimated.

## CONCLUSION

This study supports the conclusion that there is a lack of knowledge about the correct indication, type, and dosage of antibiotics in dental practice. There is also a need to improve undergraduate education and to increase the provision of postgraduate courses and other educational initiatives on antibiotic prescribing.
